# Coupling plasmonic and electron-mediated effects in Ag_*x*_@r–TiO_2_/g-C_3_N_4_ heterostructures for enhanced catalytic hydrogen generation†

**DOI:** 10.1039/d5na00267b

**Published:** 2025-07-15

**Authors:** Kashaf Ul Sahar, Khezina Rafiq, Muhammad Zeeshan Abid, Ubaid Ur Rehman, Talib K. Ibrahim, Abdul Rauf, Ejaz Hussain

**Affiliations:** a Institute of Chemistry, Inorganic Materials Laboratory 52S, The Islamia University of Bahawalpur–63100 Bahawalpur Pakistan ejaz.hussain@iub.edu.pk khezina.rafiq@iub.edu.pk; b School of Physics, State Key Laboratory of Crystal Materials, Shandong University Jinan 250100 Shandong China; c Department of Petroleum Engineering, College of Engineering, Knowledge University Erbil 44001 Iraq

## Abstract

This study presents a sustainable strategy for sunlight-driven hydrogen production *via* seawater splitting. We designed Z-scheme heterostructures using silver-decorated r–TiO_2_ and g-C_3_N_4_. These materials were synthesized *via* chemical reduction, hydrothermal treatment, and controlled calcination. The synthesized materials were characterized using advanced techniques, *i.e.* XRD and Raman analysis, which confirmed the successful integration of r–TiO_2_, metallic Ag, and g-C_3_N_4_, showing strong crystallinity and interfacial coupling. UV-vis DRS analysis depicted enhanced visible-light absorption in silver-decorated r–TiO_2_/g-C_3_N_4_ composites due to the combined effects of Ti^3+^ defects, plasmonic Ag nanoparticles, and the interfacial charge transfer between their components. Mott–Schottky analysis confirmed their n-type behaviour with optimal band alignment (−0.45 *vs.* NHE for r–TiO_2_; −1.35 *vs.* NHE for g-C_3_N_4_), promoting charge separation. SEM revealed lump-like morphology with dispersed particles, while AFM indicated distinct surface roughness. Particle size distribution ranged from 20.3 to 243.1 μm (diameter) and 30.6 to 235.6 μm (length), reflecting structural heterogeneity. Moreover, the mesoporous nature of the ternary composite was confirmed using BET. The photoreaction was conducted in a glass reactor, and the hydrogen evolution rates were monitored using GC-TCD (Shimadzu, Japan). Under optimized conditions, the recorded maximum hydrogen evolution rate was 11.76 mmol g^−1^ h^−1^ in seawater and 6.63 mmol g^−1^ h^−1^ in deionized water with 4 mg of Ag_*x*_@r–TiO_2_/g-C_3_N_4_ (2 w% Ag). The amount of hydrogen evolved over Ag_2.0_@r–TiO_2_/g-C_3_N_4_ was ∼9.56- and 6.21-fold higher than that over pristine g-C_3_N_4_ and r–TiO_2_ in seawater and ∼37.94- and 14.16-fold higher in deionized water under optimized conditions, respectively. Moreover, five-run durability tests confirmed the sustainability of the catalyst. These results suggest that Ag_*x*_@r–TiO_2_/g-C_3_N_4_ is a reliable material for advancing efficient energy conversion and fuel generation.

## Abstract 2

This study demonstrates a sustainable photocatalytic approach for hydrogen production *via* seawater splitting using Ag_*x*_@r-TiO_2_/g-C_3_N_4_ heterostructures.

## Graphical abstract

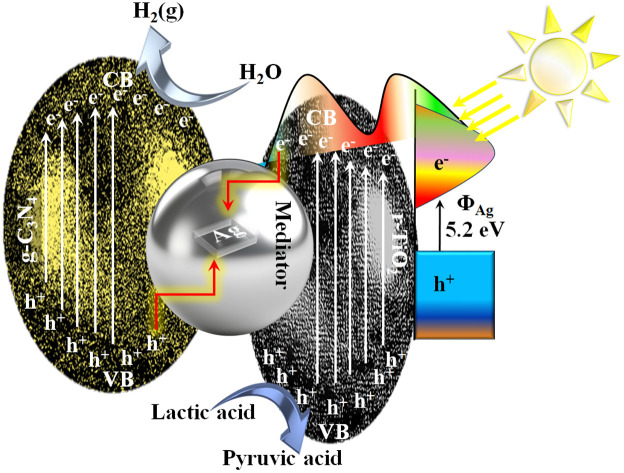

## 1. Introduction

Environmental pollution and energy storage are driving our planet toward a critical situation.^1^ Solar water splitting is an innovative process that utilizes sunlight to produce hydrogen as a clean and renewable energy.^2^ Being among the most readily available resources, the sun and seawater offer immense potential for sustainable hydrogen production.^3^ Solar energy can efficiently drive photocatalytic reactions to split water into hydrogen and oxygen, utilizing seawater as a sustainable and abundant alternative to freshwater.^4^ Although the complex composition of seawater presents some challenges, it offers an opportunity to enhance reaction efficiency with suitable photocatalysts.^5^ Seawater is composed of various ions such as sodium (Na^+^), chloride (Cl^−^), magnesium (Mg^2+^), calcium (Ca^2+^), sulphate (SO_4_^2−^), and potassium (K^+^), each contributing to its unique chemical makeup and conductivity. These ions play an important role in environmental and biological systems.^6^ Sunlight-driven hydrogen evolution using photocatalysts is a reliable approach toward a green future. Since Fujishima and Honda introduced water splitting *via* TiO_2_, numerous photocatalysts have been developed to enhance the efficiency of hydrogen production and broaden its applications. A wide range of advanced materials, including 2D materials,^7^ TiO_2_,^8^ CdS,^9^ MoS_2_,^10^ MXenes,^11^ perovskites,^12–17^ MOFs,^18^ COFs,^7^ carbon nitride,^19^ graphene^20,21^ and POMs,^22^ have demonstrated exceptional potential across diverse solar energy-driven applications, owing to their unique structural, electronic, and catalytic properties. In 2024, various advanced hybrid photocatalysts have been reported in different studies, *i.e.*, MTCPPOMe/P-MnxCd_1−*x*_S,^23^ NiFeLDH-Rh,^24^ Al_2_CO/SiC,^25^ Mg_1−*x*_Ni_*x*_Ga_*y*_Fe_2−*y*_O_4_,^26^ CuO/ZnO/graphene,^27^ Bi_0.5_Na_0.5_TiO_3_/RGO-Co_3_O_4_,^28^ Pd_0.03_/ZIS,^29^ CeO_2_/PPy/BFO,^30^ 5.9RT-SN,^31^ and metal oxide/g-C_3_N_4_.^32^ Kangutkar *et al.* synthesized MoO_3_@f-MWCNT and MoO_3_@g-C_3_N_4_ metal oxide/g-C_3_N_4_ nanocomposites, achieving H_2_ evolution rates of 2530.35 and 2313.56 μmol g^−1^ h^−1^ in DI water and 2845.06 and 2632.20 μmol g^−1^ h^−1^ in natural seawater, respectively. MoO_3_@g-C_3_N_4_ showed a superior performance with a lower Tafel slope (59 mV dec^−1^), indicating better charge transfer and interfacial synergy for efficient hydrogen generation.^33^ Researchers have explored a range of advanced catalysts for water splitting reactions; however, achieving a reliable high generation rate of hydrogen is still a challenge because each new catalyst comes with its own set of limitations. Titanium dioxide (TiO_2_) is widely documented as a promising and thermodynamically efficient photocatalyst due to its favourable cost, optical/electrical properties, excellent hydrophilicity, and remarkable durability over time. Structurally, its valence band arises from the overlap of the oxygen 2p orbitals, while its conduction band is influenced by the 3d orbitals of Ti^4+^ ions. However, its high bandgap (∼3.0 eV) limits its activation in the UV region, which constitutes only about 4% of sunlight.^34–36^ Thus, over the years, researchers have introduced numerous innovative approaches to expand its light absorption range and enhance its photocatalytic efficiency, including surface modification,^37^ heterojunction fabrication,^38^ metal oxide coating,^39^ plasmonic metal loading,^40^ anion/cation doping,^41^ core–shell microsphere structures,^42^ carbon-based modification,^43^ vacuum/plasma treatment^44^ and multidimensional architectures.^45^ These methods significantly boost the photocatalytic capabilities of TiO_2_ by reducing photogenerated charge (e^−^/h^+^) recombination, especially when doped with metallic (*e.g.*, Cr, Fe, and V) and non-metallic elements (*e.g.*, N, C, and B), which create active sites and enhance the hydrogen production efficiency.^46^ Graphitic carbon nitride (g-C_3_N_4_), a 2D carbon–nitrogen polymer with a bandgap (*E*_g_) of about ∼2.7 eV (at 460 nm), has also attracted considerable attention in photocatalysis. Owing to its high conductivity, dielectric strength, and impressive photocatalytic activity, g-C_3_N_4_ is a favoured material due to its cost-effectiveness, thermal stability (up to ∼600 °C), and distinct layered structure.^47–49^ However, its inherent challenges such as structural disorder, poor dispersibility, and low processability can limit its potential. Alternatively, heterojunction formation involves coupling two semiconductors with complementary electronic and optical properties to overcome the limitations of individual materials, such as restricted light absorption and high e^−^/h^+^ recombination rates.^50–52^ Furthermore, specific band alignments can be achieved through heterojunctions such as type I, type II, p–n, Z-scheme, and S-scheme, enhancing the charge separation and transfer efficiency.^53^ The enhanced performance is due to the improved charge transfer, increased surface area, and better hydrophilicity, which promote photocatalytic H_2_ evolution.^54–56^ This synergy between materials in heterojunctions optimizes their photocatalytic activity by leveraging the strengths of each component.^57,58^ The g-C_3_N_4_/TiO_2_ junction overcomes the limitations of each material by forming a Z-scheme heterojunction at their interface, which effectively decreases the charge recombination and improves the photocatalytic efficiency.^59–61^ The well-aligned interface within this heterojunction provides pathways for photogenerated charges to move freely, enabling greater charge separation and transport across the catalyst.^62^ To enhance the efficiency of the g-C_3_N_4_/TiO_2_ photocatalytic system, cocatalysts such as Pd, Pt, Au, Cu, Ni, and Ag have been commonly used,^63^ while Ag plays a key role due to its effective surface plasmon resonance (SPR) effect and ability to act as an electron mediator.^64–67^ The SPR effect of Ag broadens the light absorption into the visible range, increasing the overall light-harvesting efficiency of the system.^68,69^ It also facilitates electron transfer between the conduction bands (CB) of g-C_3_N_4_ and TiO_2_, reducing the charge recombination and stabilizing the charge carriers. This electron mediation enhances the photocatalytic activity of the system, improving its overall performance. Transition metals such as Cu, Ni, and Au enhance the photocatalytic activity by quenching electrons and introducing active sites. Among them, Ag is particularly effective due to its unique combination of surface plasmon resonance and electron mediation.^70–73^

In this research, we synthesized Ag_*x*_@r–TiO_2_/g-C_3_N_4_ catalysts *via* different approaches including chemical reduction and hydrothermal treatment, followed by calcination and acquired the optimized morphology. During this process, Ag nanoparticles were *in situ* loaded onto the r–TiO_2_/g-C_3_N_4_ composite. The introduction of Ag enhanced the photocatalytic performance through its unique SPR effect, which extended the light absorption of the composite into the visible range, increasing its photon utilization. Additionally, Ag acted as an effective electron mediator, facilitating rapid charge transfer between the r–TiO_2_ and g-C_3_N_4_ components, improving the charge separation and reducing recombination losses. Lactic acid was employed as a sacrificial reagent, enhancing the rate of the photocatalytic hydrogen evolution reaction by reducing charge recombination. The crystallinity, phase purity, vibrational modes, morphology, nanoparticle diameter/length, surface area and porosity of the catalysts were thoroughly characterized to understand the key factors driving their enhanced photocatalytic activity. Furthermore, factors including dose, pH, light intensity, and temperature were thoroughly optimized in two water media (seawater and deionized water (DI-H_2_O)). The synthesised ternary system maintained high efficiency in both deionized and seawater due to the synergy among r–TiO_2_, g-C_3_N_4_ and Ag nanoparticles, which optimized the electron transfer and minimized the charge recombination. Thus, the Ag_*x*_@r–TiO_2_/g-C_3_N_4_ system demonstrated significant promise for hydrogen generation, offering great potential as a solution to critical issues related to the energy and environmental crises.

## 2. Experimental

The chemicals required and the characterizations tools utilized in the current studies are described in the (ESI†).

### 2.1. Synthesis of Ag@r–TiO_2_/g-C_3_N_4_ photocatalysts

**Fig. 1:**
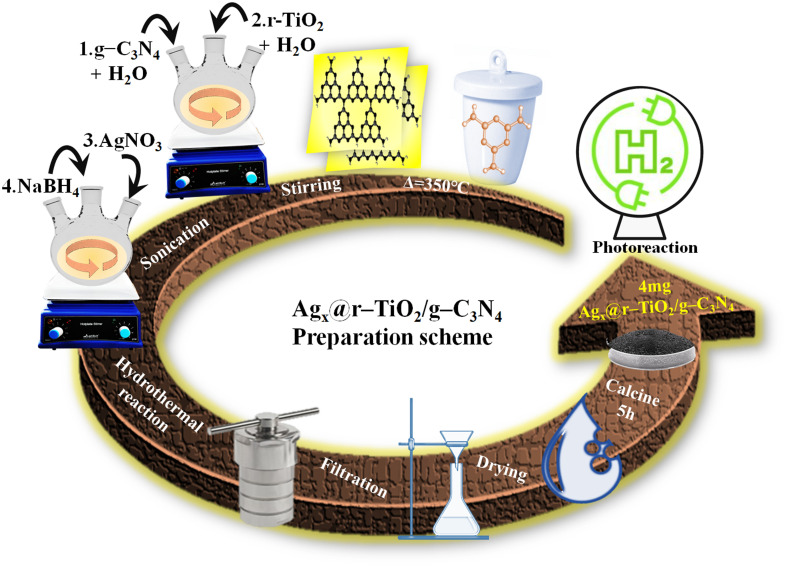
Preparation scheme for Ag_*x*_@r–TiO_2_/g-C_3_N_4_ photocatalysts.

Primarily, graphitic carbon nitride (g-C_3_N_4_) was synthesized *via* the solid-state thermal-polycondensation of melamine. Then, 50 mg of synthesized g-C_3_N_4_ was added to a three-neck round-bottom flask containing 10 mL of deionized water and stirred for 2 h followed by sonication (10 min). After that, 50 mg of rutile TiO_2_ (r–TiO_2_) and 10 mL of water were added to the flask. To load silver metal onto the surface of r–TiO_2_/g-C_3_N_4_, silver nitrate (prepared solution) was introduced in the flask containing the g-C_3_N_4_ suspension and sonicated (2 min). Then, 30 mg of sodium borohydride was dissolved in a small volume of deionized water, and 3 drops of the solution added to the flask. A change in colour (yellowish → grey) and generation of bubbles in the suspension were observed. The suspension was subjected to hydrothermal treatment in a Teflon-lined autoclave at 150 °C/2 h and the resulting solid was filtered, followed by drying overnight at 80 °C. Then the synthesized Ag_*x*_@r–TiO_2_/g-C_3_N_4_ was subjected to post-hydrothermal calcination at 350 °C for 5 h. The synthesis scheme for Ag_*x*_@r–TiO_2_/g-C_3_N_4_ is depicted in Fig. 1.

### 2.2. Optimized hydrogen generation experimentation

Hydrogen generation experiments were conducted in a 140 mL sealed glass reactor under concentrated natural sunlight (photon flux: 6.5 mW cm^−2^), according to eqn (1) and (2).1
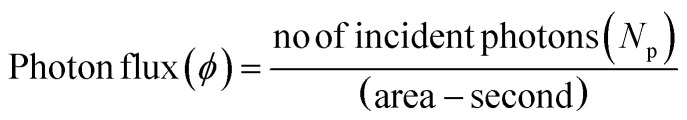
2
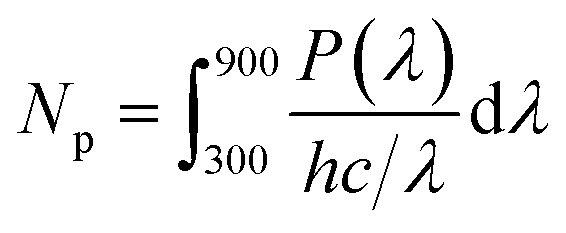
where *P*(*λ*) represents the spectral irradiance, *h* is Planck's constant, *c* is the speed of light in vacuum, and *d* is differential wavelength element. In each test, 4 mg of the synthesized photocatalyst was dispersed in water. Prior to illumination, the reactor was purged with pure nitrogen (N_2_) gas for 30 min to eliminate residual oxygen and establish an inert atmosphere. Lactic acid was added as a sacrificial agent to enhance hole scavenging. During the reaction, gas samples were periodically withdrawn from the reactor headspace using a 0.5 mL gas-tight syringe and injected into a gas chromatograph equipped with a thermal conductivity detector (GC-TCD) for hydrogen quantification. The GC setup, including the capillary molecular sieve column and detector specifications, is detailed in the ESI (Section 1, 1.2.1).† A calibration curve was established for quantitative analysis. All photocatalytic experiments were repeated at least five times to ensure reproducibility. Hydrogen evolution rates are reported in units of mmol g^−1^ or mmol g^−1^ h^−1^. Experiments were performed using both deionized water and Arabian seawater to evaluate the activity and robustness of the photocatalysts in different water matrices. The apparent quantum efficiency (AQE) was calculated using the standard equations (eqn (3)–(3.3)), as follows:3
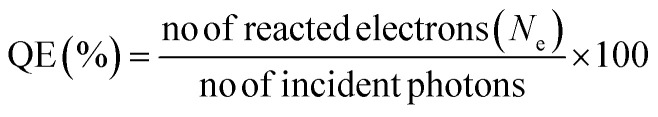
where

3.1 *N*_e_ = 2 × *n*_H_2__×*N*_A_ × *h* × *c*

In eqn (3.1), *n*_H_2__ represents the number of H_2_ molecules produced during photoreaction, *N*_A_ depicts Avogadro's number, *h* is Planck's constant and *c* represents the speed of light.3.2
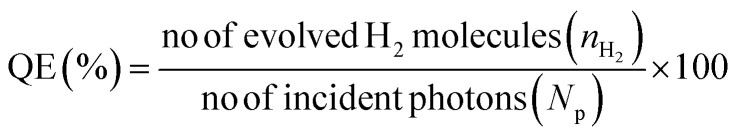
where

3.3 *N*_p_ = *S* × *P* × *t* × *λ*

In eqn (3.3), *S* represents the area of irradiation, *P* the intensity, *t* time, and *λ* depicts the wavelength of light.

Parameters such as the number of evolved hydrogen molecules, irradiation area, light intensity, reaction time, and wavelength of light were considered. The Ag_2.0_@r–TiO_2_/g-C_3_N_4_ hybrid exhibited the highest AQE of 4.1%, outperforming pristine g-C_3_N_4_, r–TiO_2_, r–TiO_2_/g-C_3_N_4_, Ag@g-C_3_N_4_, Ag@r–TiO_2_, and various Ag-loaded r–TiO_2_/g-C_3_N_4_ composites (Ag_0.5_–Ag_2.5_ series). A detailed comparison of the rate of H_2_ evolution (mmol g^−1^ h^−1^) in the current study with g-C_3_N_4_- and TiO_2_-based photocatalysts in reported studies is illustrated in Section 3, Table S7 (ESI†).

## 3. Result and discussion

### 3.1. Structural analysis and crystallinity

**Fig. 2:**
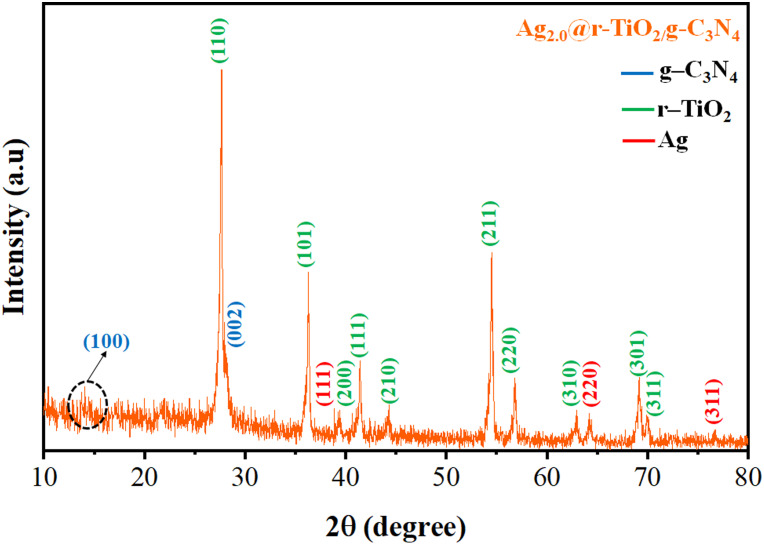
X-Ray diffraction analysis of Ag_2.0_@r–TiO_2_/g-C_3_N_4_.

X-ray diffraction (XRD) analysis was employed to investigate the crystalline structure, phase composition, and successful heterojunction formation in the synthesized Ag_2.0_@r–TiO_2_/g-C_3_N_4_ composite. The powdered XRD pattern of Ag_2.0_@r–TiO_2_/g-C_3_N_4_ is depicted in Fig. 2. The diffraction pattern was recorded in the 2*θ* range of 10° to 80°, and crystallographic phases were identified by comparison with the standard JCPDS cards. The XRD diffraction patterns of r-TiO_2_ and Ag@r-TiO_2_ are shown in ESI (see, Section 1.6 (Fig. S1†). In our prior study, the XRD analysis of bulk g-C_3_N_4_ and r–TiO_2_/g-C_3_N_4_ was thoroughly documented.^74^ The crystallite size of the nanomaterials was estimated using the Debye–Scherrer equation*D* = *Kλ*/(*β*cos*θ*), where *D* is the average crystallite size, *K* is the shape factor (0.9), *λ* is the X-ray wavelength (0.154 nm for Cu Kα), *β* is the full width at half maximum (FWHM), and *θ* is the Bragg angle. The diffraction pattern revealed multiple peaks, each corresponding to distinct crystallographic planes associated with the constituents of the composite. The g-C_3_N_4_ phase was identified by two characteristic reflections, a weak peak at approximately 13.7°, indexed to the (100) plane, representing the in-plane structural motifs of tri-*s*-triazine units, and a peak around 27.9°, attributed to the (002) plane, indicating the interlayer stacking of aromatic systems. The latter peak exhibited noticeable asymmetry and shoulder-like broadening, which was due to its overlap with the dominant (110) reflection of r–TiO_2_, indicating that both g-C_3_N_4_ and r–TiO_2_ contributed to this region. The broadened nature of the (002) peak also implied lower crystallinity or thinner stacking in the g-C_3_N_4_ domains, which is typical for this material due to its partially disordered graphitic structure (JCPDS PDF#87-1526).^75,76^ r–TiO_2_ exhibited a series of sharp and intense peaks, with, the most prominent at 27.4° corresponding to the (110) plane, a known thermodynamically stable and preferential growth facet in the rutile phase. The additional reflections at 36.1° (101), 41.2° (111), 44.0° (210), 54.3° (211), 56.6° (220), 63.0° (310), 69.0° (301), and 69.8° (311) further confirmed the presence of r–TiO_2_,^77,78^ consistent with the standard JCPDS card no. 21-1276. The broadening observed particularly in the (110) peak suggested nanoscale crystallite dimensions and possible microstrain within the lattice, both of which were corroborated by the Scherrer analysis. This broadening could also result from lattice distortions or partial reduction effects at the r–TiO_2_ surface, which are often introduced during processes for the formation of composites. Metallic silver was confirmed by weaker but distinct peaks at 38.1°, 64.4°, and 77.4°, which are assigned to the (111), (220), and (311) planes, respectively, in accordance with the face-centered cubic structure of Ag based on JCPDS card no. 04-0783. The notably low intensity of these peaks implied that silver was present in trace amounts and likely existed as highly dispersed nanoparticles with poor crystallinity/small size. These conditions tend to reduce the diffraction intensity and broaden the peaks due to the increased surface-to-volume ratio and quantum size effects typical of nanostructures. Importantly, the assignment of the (311) peak near 69.8° to TiO_2_ and the separate (311) reflection at 77.4° to Ag, based on their distinct 2*θ* positions, confirmed accurate peak labelling. Crystallite sizes were estimated using Scherrer's equation (*K* = 0.89*) after correcting for instrumental broadening. The r–TiO_2_ (110) peak at 27.4° yielded an average crystallite size of ∼20.3 nm, while the Ag (111) peak at 38.1° gave a value of ∼11.8 nm, confirming the nanoscale nature of the composite. Due to the peak overlap between g-C_3_N_4_ (002) and r–TiO_2_ (110), the g-C_3_N_4_ crystallite size was not directly calculated. However, the span of all peaks supports the presence of small crystallites in the nanocomposite. No significant peak shifting was observed, suggesting that no major lattice substitution or loading occurred; however, minor shifts could have been obscured by the inherent peak broadness. The XRD analysis validated the successful integration of g-C_3_N_4_, r–TiO_2_, and Ag into a composite structure. The sharp reflections of r–TiO_2_ and the characteristic but weaker features of Ag and g-C_3_N_4_ collectively revealed a nanocomposite with heterogeneous crystallinity and synergistic phase formation. They also provided critical insights into the crystallite sizes, interfacial interactions, and structural role of each component, confirming that the composite retained the desired multiphase configuration with nanoscale characteristics essential for potential photocatalytic water splitting.

### 3.2. Electronic and vibrational properties

**Fig. 3:**
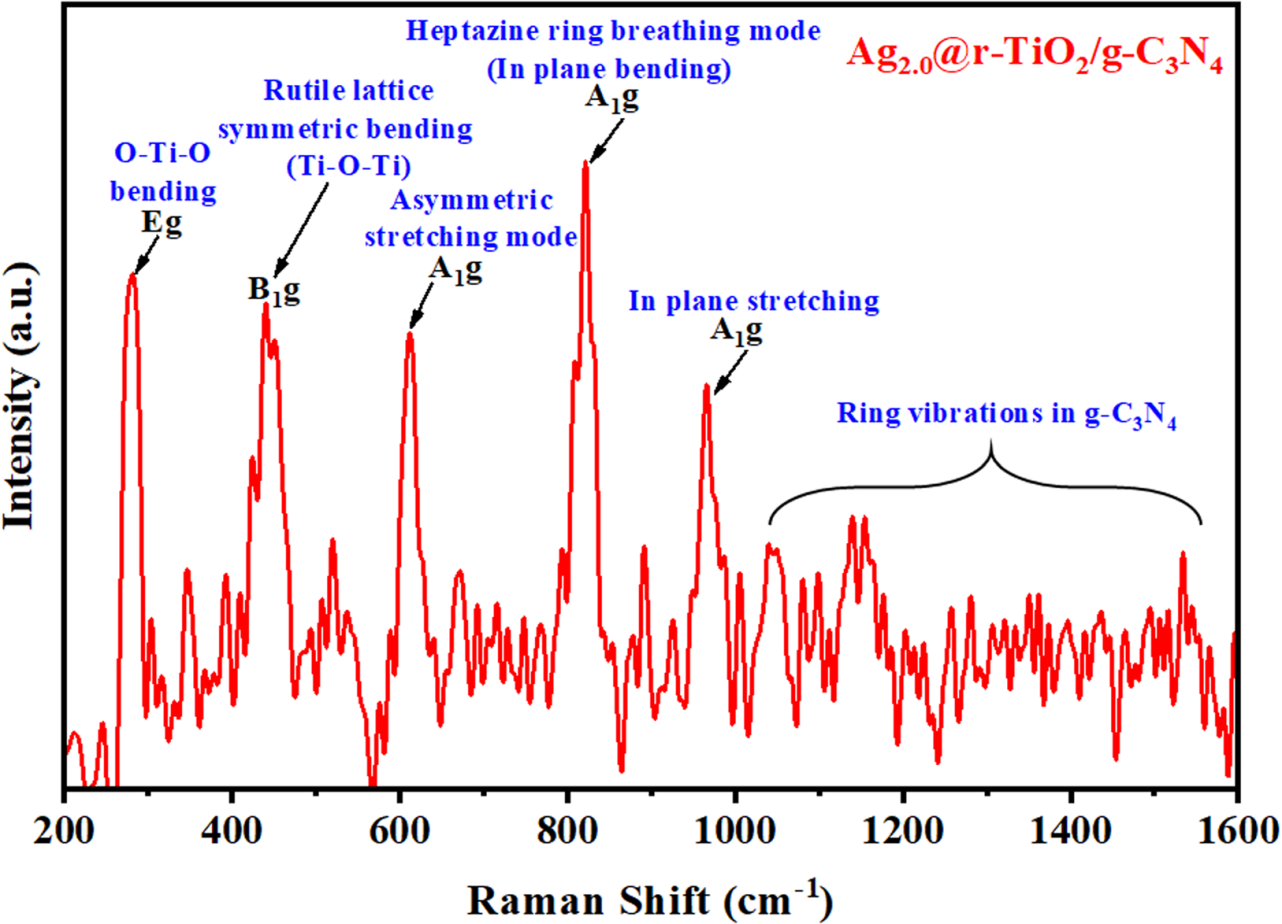
Raman spectrum of Ag_2.0_@r–TiO_2_/g-C_3_N_4_.

The Raman spectroscopic analysis of the Ag_2.0_@r–TiO_2_/g-C_3_N_4_ composite showed distinct bands in the range of 200–1600 cm^−1^, each corresponding to specific vibrational modes of r–TiO_2_ and g-C_3_N_4_, as shown in Fig. 3. In our earlier study, the Raman spectroscopic analysis of bulk r–TiO_2_, pristine g-C_3_N_4_ and g-C_3_N_4_/r–TiO_2_ was thoroughly documented.^79^ At 285 cm^−1^, a peak labelled *E*_g_ was perceived, which is attributed to the bending vibration of the O–Ti–O bond in the structure of r–TiO_2_. The band at 420 cm^−1^, known as B_1g_, corresponds to the Ti–O–Ti symmetric bending in the rutile lattice of TiO_2_. The peak at 609 cm^−1^ represents the A_1g_ asymmetric stretching vibration of the Ti–O bond in rutile TiO_2_.^80^ In the case of g-C_3_N_4_, its Raman spectrum showed a peak at 810 cm^−1^, associated with the A_1g_ mode, which is related to the in-plane bending of the heptazine ring and is a key feature of the g-C_3_N_4_ structure. Another prominent peak appeared at 969 cm^−1^, also an A_1g_ mode interrelated to the in-plane stretching of the heptazine ring.^81^ A series of peaks associated with the vibration of the heptazine ring was observed when the spectrum extended beyond 1000 cm^−1^ up to 1600 cm^−1^. These peaks are primarily due to the ring vibration modes of the heptazine structure, which are characteristic of g-C_3_N_4_ and indicate the structural integrity of the material. Moreover, the influence of silver (Ag) on the spectrum can be understated due to its metallic characteristics, which generally do not display pronounced intrinsic Raman-active modes. Nevertheless, the incorporation of Ag in the Ag_2.0_@r–TiO_2_/g-C_3_N_4_ composites could affect their Raman spectrum in various ways. A significant effect is the SPR phenomenon, where the collective oscillation of conduction electrons on the surface of silver amplifies the Raman signal of adjacent molecules through surface-enhanced Raman scattering (SERS).^82^ Thus, the Raman analysis demonstrated the successful integration of r–TiO_2_ and g-C_3_N_4_ in the composite with well-preserved vibrational modes from both semiconductors, which corresponded well with the XRD analysis.

### 3.3. Optical properties

**Fig. 4:**
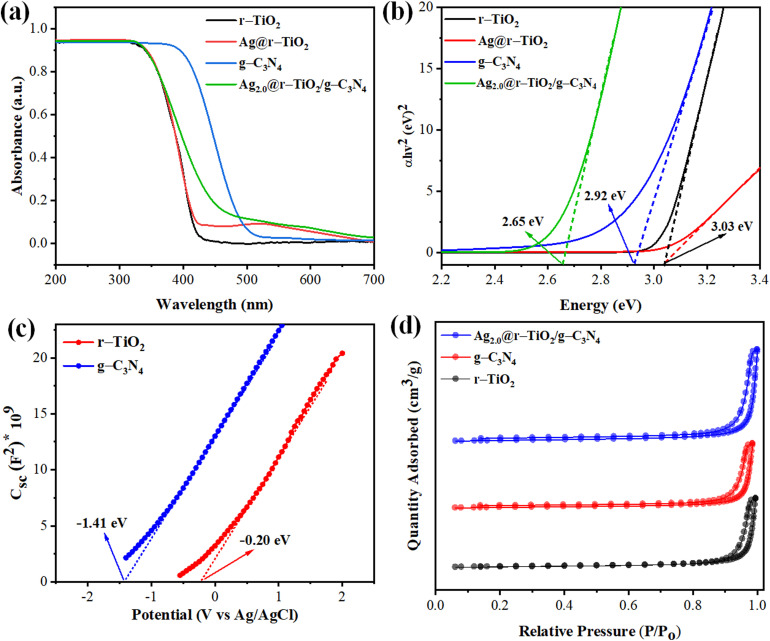
(a and b) UV-vis diffuse reflectance spectra (DRS) and Tauc plots for optical band gap determination of r–TiO_2_, Ag@r–TiO_2_, g-C_3_N_4_, and Ag_2.0_@r–TiO_2_/g-C_3_N_4_ photocatalysts; (c) Mott–Schottky plots of r–TiO_2_ and g-C_3_N_4_; (d) N_2_ adsorption–desorption isotherms of r–TiO_2_, g-C_3_N_4_, and Ag_2.0_@r–TiO_2_/g-C_3_N_4_.

The UV-DRS spectra revealed distinct light absorption characteristics for each catalyst (see, Fig. 4a), highlighting the effects of defect engineering and plasmonic coupling. The r–TiO_2_ sample exhibited an absorption edge at ∼410 nm, corresponding to a narrowed bandgap, in contrast to pristine anatase TiO_2_. This redshift was attributed to the introduction of Ti^3+^ defects and oxygen vacancies during its reduction, which created mid-gap states and enhanced visible-light absorption. The Ag@r–TiO_2_ sample retained the defect-mediated absorption edge of r–TiO_2_ (∼410 nm) but also displayed a broad plasmonic peak at ∼450–500 nm, characteristic of the localized surface plasmon resonance (LSPR) effect from metallic Ag nanoparticles. This LSPR effect significantly extended the light absorption to the visible range, which is crucial for solar-driven photocatalysis. Meanwhile, g-C_3_N_4_ showed a well-defined absorption edge at ∼460 nm, consistent with the π → π* electronic transitions in its conjugated tri-*s*-triazine structure.^83^ The ternary Ag_2.0_@r–TiO_2_/g-C_3_N_4_ composite demonstrated synergistic absorption behaviour, combining the optical properties of all its components. The r–TiO_2_ defect-related edge (∼410 nm) and g-C_3_N_4_ visible absorption (∼460 nm) were preserved, while the plasmonic Ag nanoparticles contributed a broad LSPR band (∼450–550 nm). Additionally, the presence of a slight absorption tail beyond 550 nm suggested interfacial charge transfer (ICT) among r–TiO_2_, g-C_3_N_4_ and Ag, further enhancing the visible-light utilization. The Tauc plot analysis confirmed these observations, as depicted in Fig. 4b. The bandgap values were calculated using the Kubelka–Munk function [(*αhν*)^2^*vs. hν*] for direct transitions. The r–TiO_2_ sample exhibited a bandgap of ∼3.03 eV, while g-C_3_N_4_ showed a narrower bandgap of ∼2.65 eV. The Ag_2.0_@r–TiO_2_/g-C_3_N_4_ heterostructures displayed an intermediate bandgap (∼2.92 eV), indicating electronic interactions among its components. The slight red shift compared to that for pure r–TiO_2_ confirmed the successful coupling of g-C_3_N_4_ and the plasmonic effect of Ag, which modified the electronic structure and enhanced the charge carrier generation under visible light. The combination of SPR effects from Ag, extended π-conjugation in g-C_3_N_4_, and interfacial charge transfer at the heterojunction enables this broadened optical response.

Furthermore, Mott–Schottky measurements were performed to determine the flat-band potentials and semiconductor nature of the photocatalysts. The positive slope of the linear region for r–TiO_2_ and g-C_3_N_4_ confirmed their n-type semiconductor behaviour. The flat-band potentials found to be −0.25 V *vs.* Ag/AgCl (corresponding to −0.45 V *vs.* NHE) for r–TiO_2_ and −1.15 V *vs.* Ag/AgCl (−1.35 V *vs.* NHE) for g-C_3_N_4_. This configuration shown in the CB of g-C_3_N_4_ (−1.35 V) is sufficiently negative for proton reduction, while the VB of r–TiO_2_ (+2.58 V) maintains a strong oxidative potential. The ternary Ag_2.0_@r–TiO_2_/g-C_3_N_4_ composite displayed an intermediate flat-band potential, suggesting efficient interfacial charge transfer among its components. This alignment facilitated the separation of photogenerated electrons and holes, thereby enhancing the photocatalytic hydrogen evolution.

### 3.4. Morphology

**Fig. 5:**
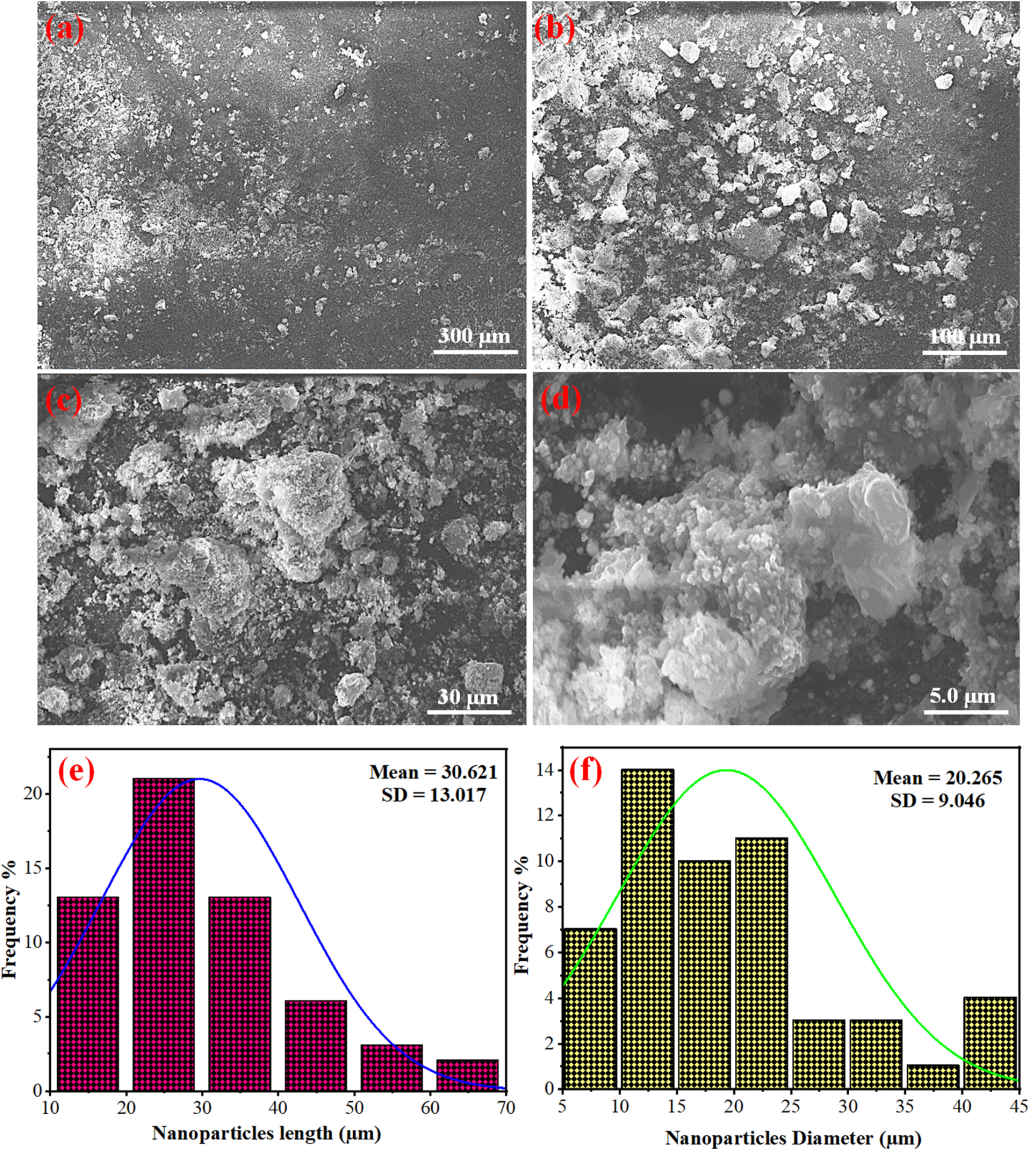
SEM images of Ag_2.0_@r–TiO_2_/g-C_3_N_4_ (most active photocatalyst in the series) at (a) 300 μm, (b) 100 μm, (c) 30 μm, and (d) 5 μm; nanoparticle size distribution in terms of (e and f) length and diameter (μm).

Scanning electron microscopy (SEM) was employed to investigate the morphology and structural features of the Ag_2.0_@r–TiO_2_/g-C_3_N_4_ photocatalyst, which was identified as the most active material in the series. SEM imaging was conducted at various scales of 300 μm, 100 μm, 30 μm, and 5 μm to capture a comprehensive view of the structural association in the synthesised material, as illustrated in Fig. 5a–d, respectively. The images revealed well-defined lump-like particles further decorated with smaller particles, likely representing g-C_3_N_4_ containing r–TiO_2_. This morphology indicated a orb-like well-integrated composite structure, with smaller particles dispersed over larger agglomerates, suggesting a high surface area and enhanced interface interactions, which can contribute to improved photocatalytic activity. Further, to quantify the particle size distribution, the ImageJ software was applied using the 100 μm scale image for accurate measurements. We measured the length and diameter distributions of the particles and represented their frequency across different size ranges using histograms Fig. 5e and f. The particle diameter examination revealed mean values ranging from 20.3 μm to 243.1 μm, with standard deviations between 9.0 μm and 43.5 μm. The nanoparticle length exhibited mean values in the range of 30.6 μm to 235.6 μm, with standard deviations of 13.0 μm to 40.0 μm. The significant variations in particle size, from minimum to maximum, highlighted the heterogeneous nature of the composite structure. These findings highlighted the structural complexity of the photocatalysts, where the particle size and distribution directly resulted from the synthesis process and the incorporation of multiple components. The combination of SEM imaging and ImageJ analysis provided critical insights into the material architecture by linking its morphology to potential performance enhancements. The observed well-defined particle interfaces and distribution underscore the suitability of this material for photocatalytic hydrogen evolution, where the optimal particle arrangement and surface interactions are key to efficient catalytic processes.

### 3.5. Surface topography

**Fig. 6:**
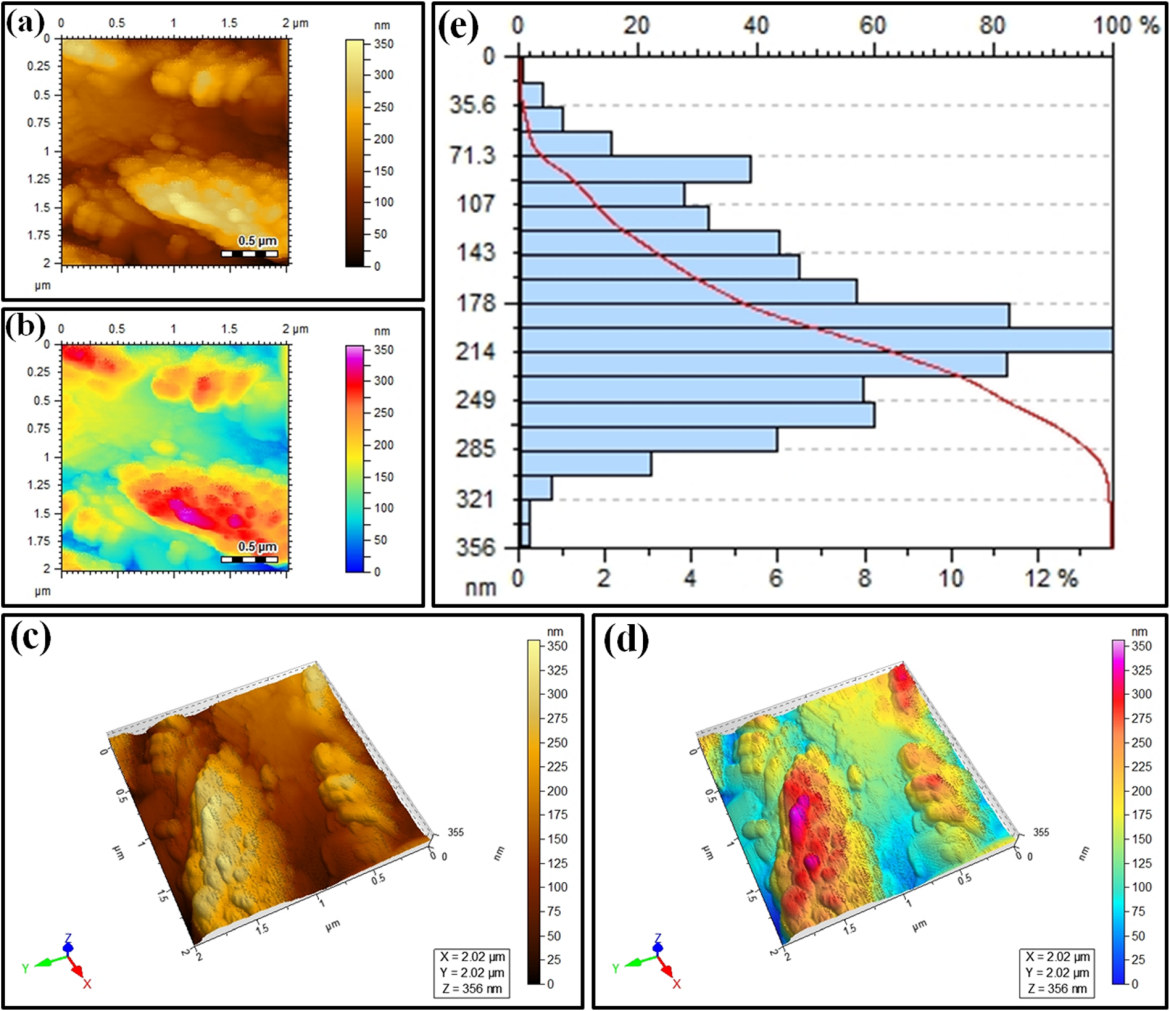
(a and b) AFM surface topography *via* single-tone brown scale and pseudo-colour height map; (c) 2D single-tone brown scale; (d) 3D pseudo-colour height map; (e) height distribution profile.

Atomic force microscopy (AFM) was performed on the Ag_2.0_@r–TiO_2_/g-C_3_N_4_ photocatalyst to investigate its surface topography and roughness. 2D and 3D images were obtained in brown gradient and pseudo-colour map, respectively. Fig. 6a and b. depict the 2D topographies at 0.5 μm, while Fig. 6c and d represent the 3D images of Ag_2.0_@r–TiO_2_/g-C_3_N_4_. In the height map, the variations in shades indicate differences in surface elevation. Lighter areas correspond to raised features or peaks, while darker areas represent depressions or valleys. The surface morphology of the photocatalyst, including its texture and uniformity, was clearly illustrated in this visualization. The pseudo-colour height map uses a gradient of colours to depict height variations more vividly, aiding in the identification of specific surface features and providing a clearer understanding of the material roughness, as shown in Fig. 6b. Fig. 6e presents the AFM height distribution profile, which is often used to analyse the surface roughness or morphology of materials. On the horizontal *X*-axis, the upper scale shows the height in nanometers, representing the vertical dimensions of the surface features. The lower scale shows the percentage frequency (%), reflecting how often a given height value occurs. Alternatively, the vertical *Y*-axis indicates the height bins in nanometers, representing different height ranges for the surface features. The bars show the frequency distribution of the heights, where the taller a bar, the greater the surface area corresponding to that height. The red line represents the cumulative height distribution, showing the percentage of the surface area below a specific height. This image depicts a broader distribution, indicating a rougher surface, while a narrower distribution signifies a smoother surface. The maximum surface height difference of 356 nm was reflected by the *X*-axis scale (0 to 356 nm). These results demonstrated the structural complexity of the photocatalyst with distinct roughness features and transitions that are critical to understanding its surface properties and their influence on its photocatalytic performance.

### 3.6. Porosity analysis

The textural characteristics of the photocatalysts were evaluated through N_2_ adsorption–desorption measurements at 77 K, as depicted in Fig. 4d. All the samples exhibited type IV isotherms with distinct H_3_-type hysteresis loops,^84^ confirming the presence of mesoporous structures with slit-shaped pores, consistent with aggregated orb-like particles. The r–TiO_2_ sample demonstrated a specific surface area of 52.3 m^2^ g^−1^, with a total pore volume of 0.18 cm^3^ g^−1^ and an average pore diameter of 6.2 nm, reflecting its moderately porous morphology. In contrast, g-C_3_N_4_ displayed a significantly higher surface area of 83.7 m^2^ g^−1^, along with a pore volume of 0.25 cm^3^ g^−1^ and a broader pore size distribution centered at 8.5 nm, which can be attributed to its layered, polymeric structure with inherent interlayer gaps. In the case of the Ag_2.0_@r–TiO_2_/g-C_3_N_4_ ternary composite, its surface area was measured to be 64.5 m^2^ g^−1^, which is intermediate among its individual components, while their pore volume (0.21 cm^3^ g^−1^) and average pore diameter (7.1 nm) were preserved. This indicated that the integration of Ag nanoparticles and g-C_3_N_4_ nanoparticles into the r–TiO_2_ framework did not cause significant pore blockage, maintaining the structural integrity of the heterostructures. The retention of mesoporosity is particularly advantageous for photocatalytic applications, given that it facilitates the following: (1) enhanced reactant adsorption (*e.g.*, H_2_O or organic molecules) due to the accessible active sites. (2) Efficient mass transport of reactants/products through interconnected pore channels. (3) Improved light penetration due to reduced scattering in the hierarchical pore network. The slight reduction in surface area compared to pure g-C_3_N_4_ (from 83.7 to 64.5 m^2^ g^−1^) was attributed to the partial embedding of Ag NPs and g-C_3_N_4_ layers within the r–TiO_2_ matrix, which optimized the trade-off between active site density and charge-carrier mobility. These BET results are consistent with the nanoscale crystallite sizes estimated based on the XRD analysis, supporting the porous and nanostructured nature of the composite. Also, the results are consistent with the data obtained from the SEM and ImageJ analysis.

## 4. Optimized rate of H_2_ evolution

**Fig. 7:**
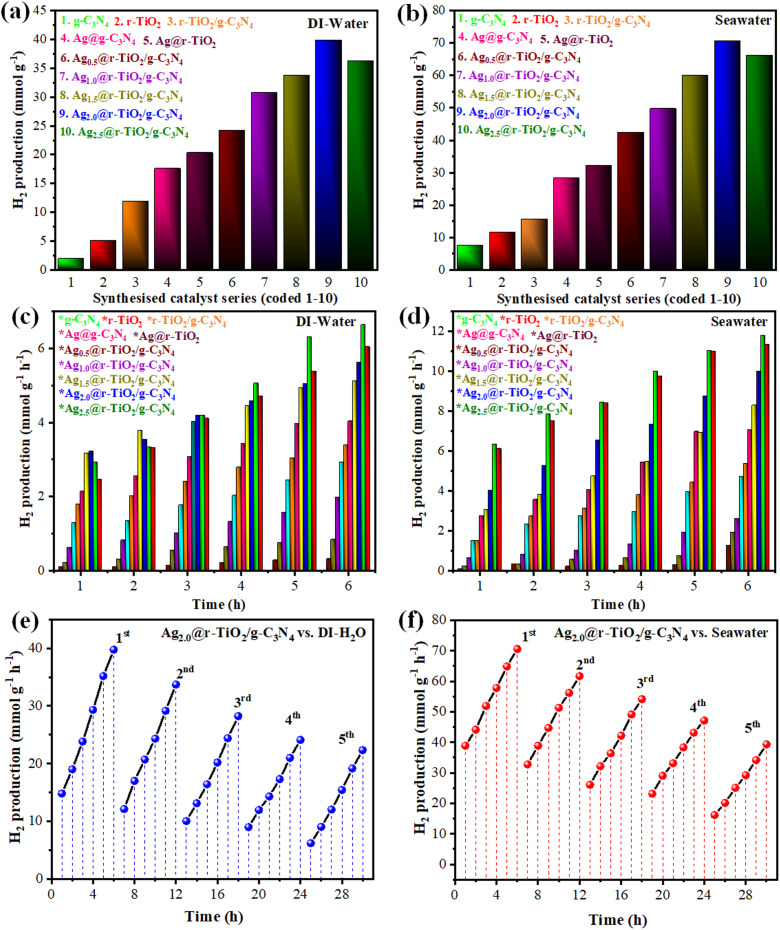
Hydrogen generation activities of bulk g-C_3_N_4_, bulk r–TiO_2_, r–TiO_2_/g-C_3_N_4_, r–TiO_2_/g-C_3_N_4_, Ag@r–TiO_2_, Ag_0.5_@r–TiO_2_/g-C_3_N_4_, Ag_1.0_@r–TiO_2_/g-C_3_N_4_, Ag_1.5_@r–TiO_2_/g-C_3_N_4_, Ag_2.0_@r–TiO_2_/g-C_3_N_4_, and Ag_2.5_@r–TiO_2_/g-C_3_N_4_ in deionized water and seawater; (a & b) mmol g^−1^ and (c & d) mmol g^−1^ h^−1^ durability test for the high-performance photocatalysts (Ag_2.0_@r–TiO_2_/g-C_3_N_4_) in (e) deionized water and (f) seawater.

In the current study, the hydrogen production efficiency of the as-synthesized photocatalysts (pristine g-C_3_N_4_, bulk r–TiO_2_, r–TiO_2_/g-C_3_N_4_, r–TiO_2_/g-C_3_N_4_, Ag@r–TiO_2_, Ag_0.5_@r–TiO_2_/g-C_3_N_4_, Ag_1.0_@r–TiO_2_/g-C_3_N_4_, Ag_1.5_@r–TiO_2_/g-C_3_N_4_, Ag_2.0_@r–TiO_2_/g-C_3_N_4_, and Ag_2.5_@r–TiO_2_/g-C_3_N_4_) were evaluated through sunlight-driven water splitting in both deionized water and seawater, as illustrated in Table 1. The comparative analysis of the hydrogen generation activities in both seawater and deionized water by all the synthesized photocatalysts was scrutinized in mmol g^−1^, as shown in Fig. 7a and b, and in mmol g^−1^ h^−1^, as shown in Fig. 7c and d. The photocatalytic activities were optimized using a catalyst dose of 4 mg for 6 h photoreaction under the optimized conditions. Bare g-C_3_N_4_ and r–TiO_2_ exhibited hydrogen evolution rates of 1.86 mmol g^−1^ and 4.98 mmol g^−1^, in DI water, which increased to 7.38 mmol g^−1^ and 11.36 mmol g^−1^ in seawater, respectively. These relatively low activities were attributed to the high charge carrier recombination and intrinsic stability limitations of the individual materials. Alternatively, the performance dramatically improved with the construction of the r–TiO_2_/g-C_3_N_4_ heterojunction, yielding 11.82 mmol g^−1^ in DI water and 15.54 mmol g^−1^ in seawater due to the enhanced charge separation. The loading of Ag nanoparticles further enhanced its photocatalytic performance. Photocatalysts such as Ag@g-C_3_N_4_ and Ag@r–TiO_2_ (both containing 2 wt% Ag) demonstrated hydrogen evolution rates of 17.52 mmol g^−1^ and 20.28 mmol g^−1^ in DI water, which increased to 28.26 mmol g^−1^ and 32.04 mmol g^−1^ in seawater, respectively. The sequentially loaded composites clearly demonstrated the synergistic effect of Ag nanoparticles on the r–TiO_2_/g-C_3_N_4_ heterojunction.

**Table 1:** Experimental hydrogen generation activities of the as-synthesized photocatalysts^a^

Sr. no.	Photocatalysts	H_2_ evolution (mmol g^−1^)		H_2_ evolution (mmol g^−1^ h^−1^)	
		DI-H_2_O seawater		DI-H_2_O seawater	
1	g-C_3_N_4_	1.86	7.38	0.31	1.230
2	r–TiO_2_	4.98	11.36	0.83	1.893
3	r–TiO_2_/g-C_3_N_4_	11.82	15.54	1.97	2.59
4	Ag@g-C_3_N_4_	17.52	28.26	2.92	4.71
5	Ag@r–TiO_2_	20.28	32.04	3.38	5.34
6	Ag_0.5_@r–TiO_2_/g-C_3_N_4_	24.18	42.30	4.03	7.05
7	Ag_1.0_@r–TiO_2_/g-C_3_N_4_	30.66	49.62	5.11	8.27
8	Ag_1.5_@r–TiO_2_/g-C_3_N_4_	33.72	59.94	5.62	9.99
9	Ag_2.0_@r–TiO_2_/g-C_3_N_4_	39.78	70.56	6.63	11.76
10	Ag_2.5_@r–TiO_2_/g-C_3_N_4_	36.24	66.06	6.04	11.31

a Optimized system includes concentrated sunlight irradiation, 4 mg dose of photocatalysts; pH = 8.0; temperature = 35 °C.

The Ag_2.0_@r–TiO_2_/g-C_3_N_4_ catalyst emerged as the most efficient, achieving 39.78 mmol g^−1^ in DI water and 70.56 mmol g^−1^ in seawater, corresponding to hydrogen evolution rates of 6.63 mmol g^−1^ h^−1^ and 11.76 mmol g^−1^ h^−1^, respectively. Here, the Ag nanoparticles acted as efficient electron mediators, facilitating the rapid transfer of photogenerated electrons from the conduction band of the semiconductors to the reaction sites, thereby suppressing charge recombination. The SPR effect from the Ag nanoparticles greatly enhanced the visible light harvesting and intensified the photocatalytic reaction. The consistent increase in performance with different Ag loadings highlighted the key role of Ag dosage in achieving a superior catalytic function. Interestingly, seawater showed higher hydrogen evolution rates compared to DI water across all samples. This enhancement is attributed to the ionic species in seawater that potentially interact with the photocatalyst surface, further boosting the charge separation and reaction kinetics. The results confirm the potential of Ag_2.0_@r–TiO_2_/g-C_3_N_4_ as a highly efficient and scalable photocatalyst for hydrogen production.

### 4.1. Durability test

In the present research, Ag_2.0_@r–TiO_2_/g-C_3_N_4_ was identified as a highly efficient photocatalyst by demonstrating exceptional activity for hydrogen generation under ambient conditions. The recyclability of this photocatalyst was tested in over five runs, totalling 30 h of reaction time. The results are summarized in Table S1 and S2† and illustrated in Fig. 7e and f, showing remarkable stability with only a minor reduction in performance across successive cycles. In the durability testing, the reactions were maintained continuously for 6 h per experimental run. The photocatalyst was retrieved by centrifugation after the completion of each cycle and thoroughly washed with deionized water to remove any residual reactants. Then, the washed photocatalyst was dried at 80 °C to prepare it for the next reaction. This regeneration process ensured that Ag_2.0_@r–TiO_2_/g-C_3_N_4_ remained active and reusable for multiple rounds. This quantitative analysis revealed <5% reduction in hydrogen evolution rate per cycle, confirming the exceptional structural preservation and functional retention in the photocatalyst. This durability highlighted the robustness of Ag_2.0_@r–TiO_2_/g-C_3_N_4_ for practical applications. The ability to maintain activity over extended use underscores the potential of Ag_2.0_@r–TiO_2_/g-C_3_N_4_ as a sustainable and cost-effective solution for the generation of clean energy. Further investigations into its long-term stability and optimization of its regeneration protocols can augment its performance in the future.

### 4.2. Plausible reaction mechanism

The photocatalytic H_2_ evolution reaction (HER) and O_2_ evolution reaction (OER) are vital in the water splitting process. In this case, for the photocatalyst to drive these reactions efficiently, its conduction band (CB) potential must be more negative than the hydrogen reduction potential (0 V *vs.* NHE), and its valence band (VB) potential must be more positive than the oxygen oxidation potential (+1.23 V *vs.* NHE). Single semiconductors often face limitations in achieving these requirements due to their rapid e^−^/h^+^ recombination and insufficient redox potentials. Alternatively, the ternary system (Ag_2.0_@r–TiO_2_/g-C_3_N_4_) investigated in our study operated under a direct Z-scheme mechanism, where Ag nanoparticles mediated the transfer of photogenerated electrons between r–TiO_2_ and g-C_3_N_4_. Upon exposure to sunlight, both g-C_3_N_4_ and r–TiO_2_ absorbed photons, generating electron–hole pairs, according to eqn (4) and (5).

4 g−C_3_N_4_ + *hν* → e^−^_CB_(g−C_3_N_4_) + *h*^+^_VB_(g−C_3_N_4_)

5 r−TiO_2_ + *hν* → e^−^_CB_(r−TiO_2_) + *h*^+^_VB_(r−TiO_2_)

The strategic role of Ag nanoparticles is to enhance the charge transfer and reduce charge recombination by facilitating electron movement from the CB of r–TiO_2_ to the CB of g-C_3_N_4_, as well as acting as a plasmonic enhancer under visible light. Under light irradiation (*hν*), the surface plasmon resonance (SPR) effect of the Ag nanoparticles generates energetic electrons and induces a localized electromagnetic field (EMF), which facilitate charge separation and accelerate interfacial electron transfer. Through SPR, Ag generates hot electrons, which are injected into the CB of r–TiO_2_, increasing the overall efficiency of the photocatalytic process. The interaction between Ag and the photocatalyst leads to the following reactions, as shown by eqn (6) and (7):

6 Ag_SPR_ + *hν* → e^−^_hot_ + h^+^_Ag_

7 e^−^_hot_ → e^−^_CB_(r−TiO_2_)

Specifically, the electrons are excited from the VB to the CB of both materials, leaving behind a hole in the VB. The CB and VB potentials of g-C_3_N_4_ and r–TiO_2_ enable efficient charge transfer *via* the Ag nanoparticles, facilitating the recombination of electrons from the CB of r–TiO_2_ and holes from the VB of g-C_3_N_4_. Between the junction of g-C_3_N_4_ and r–TiO_2_, Ag acts as an electron mediator, where the holes from the VB of g-C_3_N_4_ and the electrons from the CB of r–TiO_2_ are directly consumed by it. This scheme maintains a high-energy state in the CB of g-C_3_N_4_, which subsequently drives HER. Electrons in the CB of g-C_3_N_4_ reduce protons (H^+^) in the surrounding solution to produce hydrogen gas (H_2_) according to the reaction in eqn (8), as follows:

8 H^+^ + 2e^−^_CB_(g−C_3_N_4_) → H_2_

At the same time, the photogenerated holes in the VB of g-C_3_N_4_ are consumed by lactic acid (hole scavenger) and oxidized to pyruvic acid, as shown in eqn (9).

9 CH_3_CH(OH)COOH + 2h^+^_VB_ + (g−C_3_N_4_) → CH_3_COCOOH + 2H^+^

In seawater, additional species such as chloride ions (Cl^−^), bromide ions (Br^−^), and other ions also contribute to the overall photocatalytic performance. These ions can be oxidized at the VB of g-C_3_N_4_, competing with water oxidation to form chlorine and hypochlorite (from chloride) and bromine and hypobromite (from bromide). The presence of sodium (Na^+^) and magnesium (Mg^2+^) ions in seawater influences the surface charge dynamics, slightly affecting the reaction kinetics, but the overall efficiency of hydrogen production remains high due to effective charge separation and minimal recombination facilitated by the Z-scheme mechanism. The overall water-splitting reaction under sunlight can be explained by eqn (10), as follows:

10 2H_2_O + 4*hν* → 2H_2_ + O_2_

**Fig. 8:**
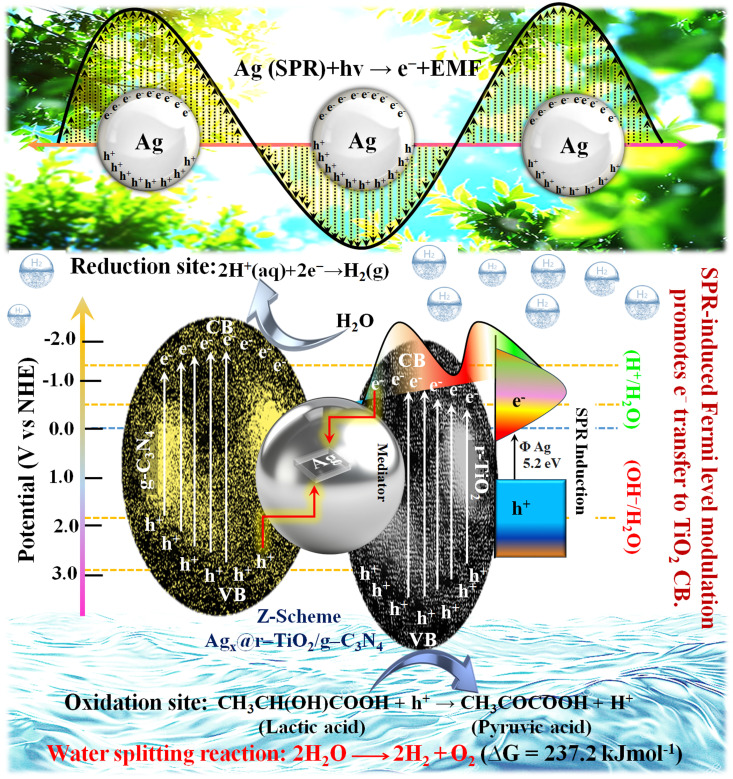
Plausible reaction mechanism of Ag_*x*_@r–TiO_2_/g-C_3_N_4_.

The synthesised ternary system maintained high efficiency in both deionized and seawater due to the synergy among r–TiO_2_, g-C_3_N_4_ and Ag nanoparticles, which optimized the electron transfer and minimized charge recombination. This Z-scheme mechanism, enhanced by SPR, ensures practical hydrogen production with the ability to utilize natural water sources such as seawater effectively. The plausible reaction mechanism by Ag_*x*_@r–TiO_2_/g-C_3_N_4_ is depicted Fig. 8.

## 5. Optimization of parameters

In the current research, parameters such as dose concentration, pH, temperature and light intensity were optimized, respectively.

### 5.1. Optimization of dose concentration

**Fig. 9:**
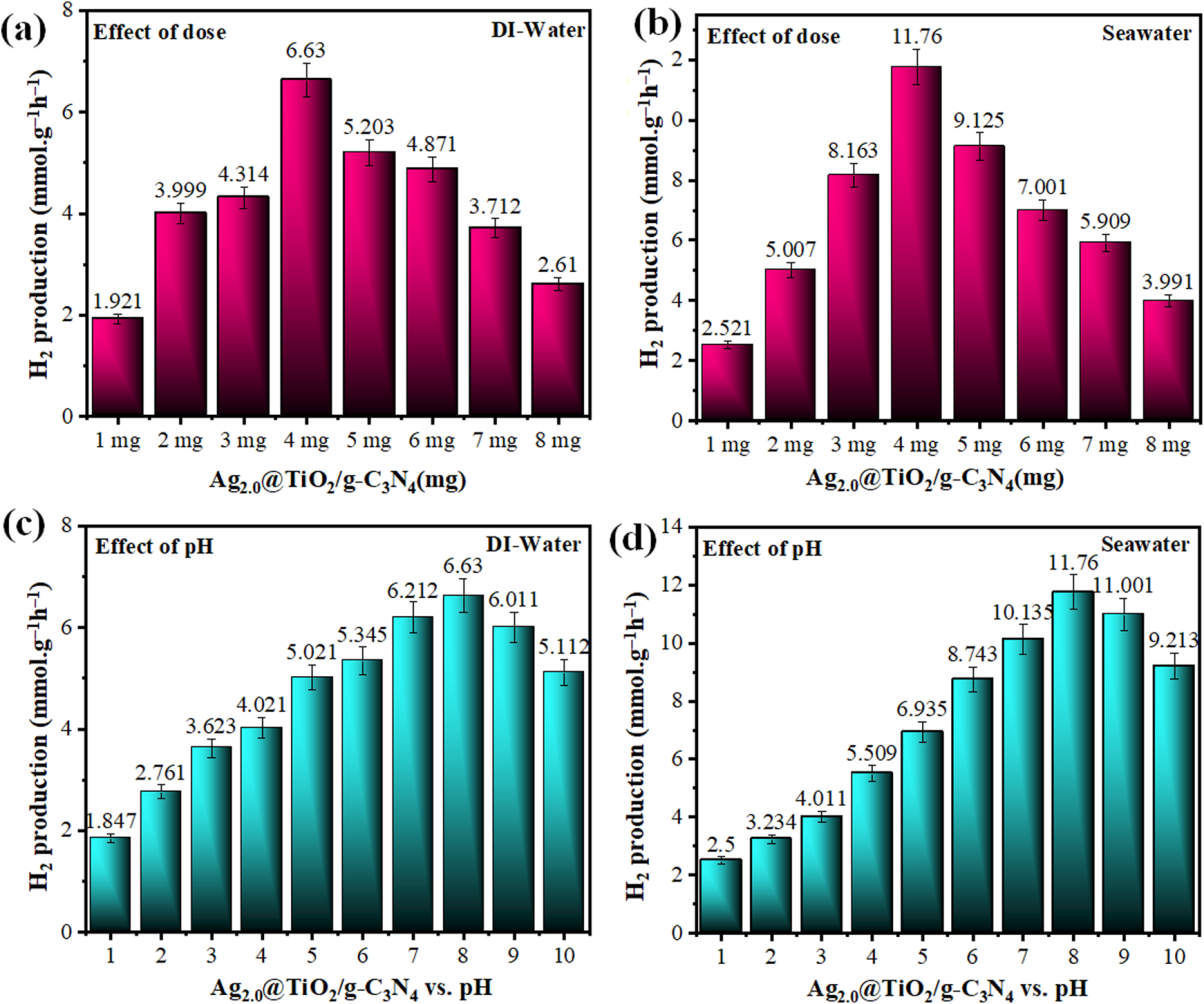
Experiments for the optimization of hydrogen generation (mmol g^−1^ h^−1^) by Ag_2.0_@r–TiO_2_/g-C_3_N_4_ (most active photocatalyst) in deionized water and seawater at different (a and b) dose concentrations and (c and d) pH.

The hydrogen evolution activity by Ag_2.0_@r–TiO_2_/g-C_3_N_4_ was systematically studied in both deionized water and seawater under varying catalyst dosages (1 to 8 mg) using concentrated sunlight irradiation. In deionized water, the photocatalyst exhibited dose-dependent behaviour, with hydrogen evolution gradually increasing to the peak of 6.63 ± 0.3315 mmol g^−1^ h^−1^ at a dose of 4 mg. The optimal concentration presumably achieves equilibrium among active site exposure, effective photon capture, and reduced particle agglomeration. Beyond 4 mg, a decline in activity was observed due to particle flocking and light scattering effects, which hindered the overall efficiency of the photocatalytic process. In seawater, the photocatalyst demonstrated superior hydrogen evolution across all doses, with the maximum of 11.76 ± 0.588 mmol g^−1^ h^−1^ also achieved at the dose of 4 mg, as shown in Table S3† and Fig. 9a and b. The enhanced activity in seawater is attributed to the interaction of halide ions with the catalyst surface, which improved the charge carrier dynamics and contributed to a synergistic effect, accelerating hydrogen production. However, similar to deionized water, higher doses beyond 4 mg led to diminished activity, further confirming that the optimal dose ensures an ideal balance between reaction kinetics and active surface availability.

### 5.2. Optimization of pH

The performance of Ag_2.0_@r–TiO_2_/g-C_3_N_4_ for hydrogen generation was studied across a range of pH levels, and the results depicted an intriguing division about the role of acidity and alkalinity in determining photocatalytic efficiency. Whether in deionized water or seawater, the photocatalyst showed an increase in hydrogen production as the environment shifted from acidic to mildly alkaline conditions, with the highest activity observed at pH 8.0 (note: HCl and NaOH were utilized to provide acidic and alkaline medium, respectively). In deionized water, hydrogen evolution began modestly at 1.847 ± 0.092 mmol g^−1^ h^−1^ at pH 1.0 and steadily improved, peaking at 6.63 ± 0.33 mmol g^−1^ h^−1^ at pH 8.0. The optimal pH range is where the balance of protons and hydroxyl ions creates the perfect conditions for efficient charge transfer, while driving HER.^85^ Interestingly, the decline in performance at higher pH is consistent with the expected proton limitations and hydroxyl competition. Notably, the seawater conditions intensified this trend, suggesting that ionic composition plays a critical role. Starting at 2.501 ± 0.125 mmol g^−1^ h^−1^ at pH 1.0, the hydrogen evolution activity increased to 11.760 ± 0.588 mmol g^−1^ h^−1^ at pH 8.0. This enhanced performance in seawater compared to deionized water is attributed to the unique environment of seawater, where ions such as chlorides support better charge separation and catalytic efficiency. Beyond pH 8.0, the trend mirrored that of deionized water, with the activity tapering off to 9.213 ± 0.4606 mmol g^−1^ h^−1^ at pH 10, as shown in Table S4† and Fig. 9c and d. These findings revealed the critical role of pH in optimizing photocatalytic systems for hydrogen production. The slightly alkaline conditions around pH 8.0 emerged as the most favourable, making Ag_2.0_@r–TiO_2_/g-C_3_N_4_ not only effective but also adaptable for real-world applications, even in challenging environments such as seawater.

### 5.3. Optimization of light intensity

**Fig. 10:**
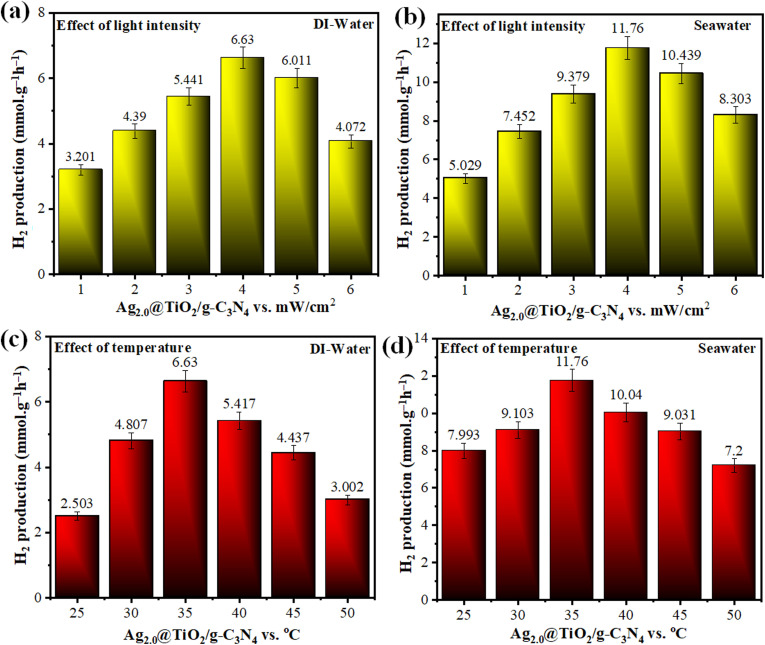
Experiments for the optimization of hydrogen generation (mmol g^−1^ h^−1^) using Ag_2.0_@r–TiO_2_/g-C_3_N_4_ (most active photocatalyst) in deionized water and seawater at different (a & b) light intensities and (c & d) temperature.

The hydrogen production performance of Ag_2.0_@r–TiO_2_/g-C_3_N_4_ was closely linked to the intensity of sunlight throughout the day by reflecting its efficient utilization of natural solar energy. Starting in the morning (10:00–11:00 AM), the photocatalyst exhibited moderate activity, producing 3.201 ± 0.160 mmol g^−1^ h^−1^ of hydrogen in deionized water and 5.029 ± 0.251 mmol g^−1^ h^−1^ in seawater. As the sunlight intensity increased, the hydrogen evolution rates steadily improved, reaching their peak during the early afternoon (1:00–2:00 PM) with 6.630 ± 0.331 mmol g^−1^ h^−1^ in deionized water and an impressive 11.76 ± 0.588 mmol g^−1^ h^−1^ in seawater. This system performed very well under the maximum sunlight intensity but showed a gradual performance decay after 2:00 PM as the irradiance decreased. Nevertheless, it retained considerable photocatalytic activity, confirming its functionality under reduced light intensity, as depicted in Table S5† and Fig. 10a and b.

### 5.4. Optimization of temperature

The photocatalytic performance of Ag_2.0_@r–TiO_2_/g-C_3_N_4_ demonstrated a strong dependence on temperature, revealing the delicate balance required for optimal hydrogen evolution. At 25 °C, the hydrogen production rates were modest, achieving 2.503 ± 0.125 mmol g^−1^ h^−1^ in deionized water and 7.993 ± 0.399 mmol g^−1^ h^−1^ in seawater. As the temperature increased to 35 °C, a notable enhancement was observed, with the rates peaking at 6.63 ± 0.331 mmol g^−1^ h^−1^ in deionized water and 11.76 ± 0.588 mmol g^−1^ h^−1^ in seawater. This increase can be attributed to the improved kinetics and charge carrier mobility at moderate temperatures. Beyond 35 °C, the hydrogen evolution began to decline, likely due to the thermal destabilization of the catalyst or competing recombination processes, which was evidenced by its reduced activity at 50 °C. The consistent performance variation under different thermal conditions established temperature regulation as a key determinant of photocatalytic efficiency, as shown in Table S6† and Fig. 10c and d.

## 6. Conclusion

In summary, this study demonstrated a sustainable approach for solar-driven hydrogen production by engineering a Z-scheme heterostructure composed of Ag-decorated r–TiO_2_ and g-C_3_N_4_. The hybrid photocatalysts, synthesized *via* chemical reduction, hydrothermal processing, and calcination, exhibited strong interfacial coupling and optimized charge transport dynamics. Among the compositions (bulk g-C_3_N_4_, r–TiO_2_, r–TiO_2_/g-C_3_N_4_, Ag@g-C_3_N_4_, Ag@r–TiO_2_, and various Ag-loaded r–TiO_2_/g-C_3_N_4_ composites (Ag_0.5_–Ag_2.5_ series)), Ag_2.0_@r–TiO_2_/g-C_3_N_4_ delivered superior hydrogen evolution, achieving 11.76 mmol g^−1^ h^−1^ in seawater and 6.63 mmol g^−1^ h^−1^ in deionized water under natural sunlight irradiation. Experimental validation through XRD and Raman spectroscopy confirmed the phase purity and effective integration of each component. UV-DRS and Mott–Schottky analyses revealed favourable band alignment, visible light harvesting, and suppressed charge recombination, corroborating the operation of a Z-scheme facilitated by Ag-mediated SPR effects. The morphology and surface analyses including SEM, AFM, and BET uncovered a mesoporous, roughened topology conducive to increased surface reaction kinetics. The synthesized composite not only outperformed pristine bulk g-C_3_N_4_ and r–TiO_2_ by significant margins (∼9.56- and 6.21-fold higher than pristine g-C_3_N_4_ and r–TiO_2_ in seawater, whereas ∼37.94- and 14.16-fold higher in deionized water under the optimized conditions, respectively) but also retained its structural integrity over multiple recycling tests, confirming its operational stability. These findings position Ag_2.0_@r–TiO_2_/g-C_3_N_4_ as a promising candidate for efficient photocatalytic hydrogen production from natural water sources, with implications for scalable clean energy technologies.

## Conflicts of interest

The author declares no competing financial interests.

## Supplementary Material

NA-OLF-D5NA00267B-s001

## Data Availability

The data and necessary protocols of this study have been included as part of the ESI.†
