# Supplementary material for: Coupling plasmonic and electron-mediated effects in Agx@r–TiO2/g-C3N4 heterostructures for enhanced catalytic hydrogen generation
Source: Nanoscale Adv. 2025 Jul 15;7(17):5384–400. doi: 10.1039/d5na00267b (PMC12308520; doi:10.1039/d5na00267b)
Supplement: NA-007-D5NA00267B-s001 [file NA-007-D5NA00267B-s001.pdf]

Supplementary Information (SI) for Nanoscale Advances.
This journal is © The Royal Society of Chemistry 2025

Supplementary Information

Coupling plasmonic and electron-mediated effects in Ag_x@r–TiO_2/g–C_3N_4 heterostructures for enhanced catalytic hydrogen generation

Kashaf Ul Sahar[a], Khezina Rafiq[a*], Muhammad Zeeshan Abid[a], Ubaid Ur Rehman[b], Talib K. Ibrahim[c], Abdul Rauf[a], Ejaz Hussain[a*]

[a]Institute of Chemistry, Inorganic Materials Laboratory 52S, The Islamia University of Bahawalpur-63100, Pakistan.

[b]School of Physics, State Key Laboratory of Crystal Materials, Shandong University, Jinan 250100, Shandong, China.

[c]Department of Petroleum Engineering, College of Engineering, Knowledge University, Erbil 44001, Iraq.

Corresponding authors: ejaz.hussain@iub.edu.pk; khezina.rafiq@iub.edu.pk

Section 1

1.1. Chemicals required

The chemicals employed in this study were sourced from trusted commercial suppliers and used without further purification. Melamine was procured from Shandong Lingmei Chemical Co., Ltd. Rutile TiO_2 from Texan Mineral and Chemicals (CAS# 1317-80-2), Silver nitrate (CAS# 7761-88-8; Sigma-Aldrich, 209139), hydrogen chloride (CAS# 7647-01-0; Sigma-Aldrich, 320331), sodium hydroxide (CAS# 1310-73-2; Sigma-Aldrich, 221465), and lactic acid (CAS# 60-00-4; Sigma-Aldrich, 69775) were all purchased from Sigma-Aldrich. Seawater samples, collected from the Arabian Sea at Karachi Port, Pakistan, were analyzed as a natural resource for the photocatalytic reactions. High-purity deionized water, essential for the experimental setup was supplied by PIAS, Pakistan. These reagents were taken based on their high purity and suitability for the experimental conditions while ensuring the integrity and reproducibility of the results.

1.2. Tools for characterization

In current study, the synthesized photocatalysts underwent comprehensive characterization to elucidate their structural, morphological, and surface traits. X-ray Diffraction (XRD) was employed to validate the crystalline structure and phase purity, with distinct peaks indicating the successful incorporation of reduced Ag nanoparticles over rutile TiO_2 and g–C_3N_4 matrix. The XRD analysis was conducted on X-ray diffractometer having Ni filtered employing Cu-Kα radiation (λ = 1.5418Å, 40 mA and 40kV). Data has been collected by using flat–plate sample holder in the Bragg−Brentano para–focusing geometry. Raman Spectroscopy facilitated the identification of vibrational modes, with significant shifts in the peaks of rutile and g–C_3N_4, suggesting robust interactions among the components, thereby enhancing the material stability.

Raman analysis of the $Ag_{2.0}$@r–TiO_2/g–C_3N_4 catalysts obtained from Horiba JY LabRAM HR 800 Raman spectrometer. Diffuse reflectance UV-Vis/DRS absorption spectra of powdered catalysts obtained from UV-Vis/DRS spectrophotometer manufactured by Thermo Fischer scientific and mounded with the praying mantis diffuse reflectance adapter. Mott–Schottky (MS) analysis was carried out using a CHI660E electrochemical workstation (CH Instruments, USA) in a standard three-electrode setup, where Ag/AgCl served as the reference electrode, Pt wire as the counter electrode, and the working electrode was prepared by coating the photocatalyst onto FTO glass. The measurements were performed in 0.5 M Na_2SO_4 solution at a frequency of 1 kHz under dark conditions. Scanning Electron Microscopy (SEM) conducted by Thermo Fisher Axia ChemiSEM, provided detailed imagery of the surface of photocatalysts, revealing a homogeneous distribution of silver nanoparticles and r–TiO_2 particles upon g–C_3N_4. Moreover, to attain a more understanding of the particle size distribution, ImageJ software was utilized to investigate the SEM images. This enabled precise measurements and statistical evaluation of the particle sizes, providing a clearer perspective on the material surface characteristics. Atomic Force Microscopy (AFM) conducted via PARK N×10 instrument illumined the surface topography, revealing a roughened surface that increases the available active area, which is advantageous for photocatalytic processes. Brunauer-Emmett-Teller (BET) study uncovered a considerable surface area with well-defined porosity, which enables efficient mass transfer during H_2 evolution. In due course, rate of H_2 evolution was quantified using a Shimadzu Gas Chromatograph with a Thermal Conductivity Detector (GC-TCD), revealing a hydrogen production rate of synthesized catalysts under sunlight in seawater. This robust performance underscores the effective synergy among Ag nanoparticles, reduced r–TiO_2, and g–C_3N_4 in fostering efficient visible light driven H_2 production.

1.2.1. Configuration of GC-TCD

The analytical measurements were conducted using a Shimadzu gas chromatography (GC) system equipped with a precision injector, temperature-programmable oven, and advanced electronic control modules for accurate thermal regulation and chromatographic performance. A specialized Shimadzu capillary column was selected to achieve optimal separation of gaseous components relevant to hydrogen production reactions, complemented by molecular sieve columns tailored for permanent gas analysis. The system incorporated high-precision gas flow controllers to maintain the carrier gas (N_2) at optimized flow rates, ensuring chromatographic resolution and reproducibility. For hydrogen detection, a high-sensitivity Thermal Conductivity Detector (TCD) was employed, leveraging its superior linear response and selectivity for light gases. The detector and column temperatures were precisely controlled using PID-regulated heating systems, with the column temperature optimized for compound-specific retention behavior and the TCD stabilized for baseline noise reduction. Chromatographic data were acquired and processed through dedicated software with customizable parameters, including sampling frequency, peak integration algorithms, and signal filtering, to enhance resolution and quantification precision. To ensure analytical validity, routine calibration protocols were implemented using certified reference standards, accompanied by system suitability tests to verify detector linearity, retention time reproducibility, and sensitivity thresholds (e.g., LOD < 0.1 ppmv for H_2).

1.3. Synthesis of r–TiO_2/g–C_3N_4

Initially, graphitic carbon nitride (g–C_3N_4) has been synthesized by solid state thermal-polycondensation of melamine. Then, 50mg of synthesized g-C_3N_4 was taken in three neck round bottom flask along containing 10mL of deionized water and stirred for 2h followed by sonication

(10 min). After that, 50mg of rutile TiO_2 along with 10mL of water was added to the flask underwent stirring (2h) followed by sonication (10 min). Then the suspension endured hydrothermal treatment in a Teflon lined autoclave at 150°C/2h, after which resulting solid was filtered and dried overnight at 80°C. Then the synthesized r–TiO_2/g–C_3N_4 underwent post hydrothermal calcination at 350°C for 5h.

1.4. Ag@g–C_3N_4

Primarily, synthesized g–C_3N_4 was taken in three neck round bottom flask along containing 10mL of deionized water and stirred for 2h followed by sonication (10 min). For silver metal loading onto synthesized g-C_3N_4 surfaces, the silver nitrate (prepared solution) was introduced to the flask containing g-C_3N_4 suspension and sonicated (2 min). Then, 30mg of sodium borohydride dissolved in a small volume of deionized water, and 3 drops of the solution added in the flask. The change in color (yellowish → grey) and origination of bubbles in the suspension were observed. The suspension endured hydrothermal treatment in a Teflon lined autoclave at 150°C/2h, after which resulting solid was filtered and dried overnight at 80°C. Then the synthesized Ag@g–C_3N_4 endured post hydrothermal calcination at 350°C for 5h.

1.5. Ag@r–TiO_2

Principally, 50mg of purchased rutile TiO_2 (r–TiO_2) was added in the three neck round bottom flask along containing 10mL of deionized water and stirred for 2h followed by sonication (10 min). For silver metal loading onto r–TiO_2 surfaces, the silver nitrate (prepared solution) was introduced to the flask containing r–TiO_2 and sonicated (2 min). Then, 30mg of sodium borohydride dissolved in a small volume of deionized water, and few drops of the solution added in the flask. The change in color (white → grey) and origination of bubbles in the suspension were observed. The

suspension endured hydrothermal treatment in a Teflon lined autoclave at 150°C/2h, after which resulting solid was filtered and dried overnight at 80°C. Then the synthesized Ag@ r–TiO_2 endured post hydrothermal calcination at 350°C/5h.

1.6. XRD Analysis

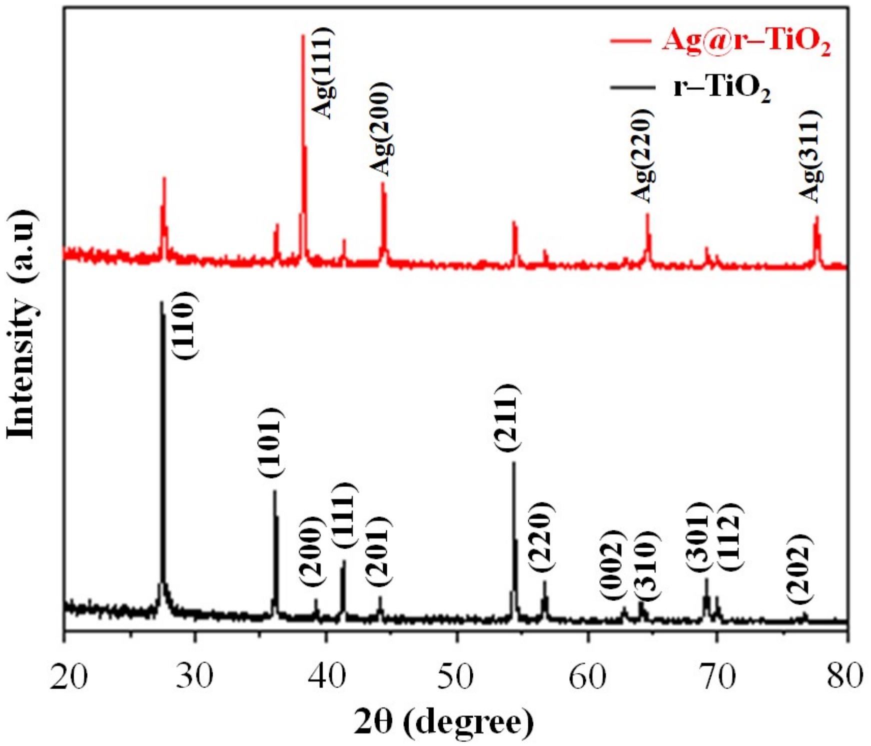

Fig. (S1). X-Ray diffraction patterns of r–TiO_2 and Ag@r–TiO_2.

Section 2

2.1. Durability test

Table. (S1). Durability test for the high performing photocatalysts ($Ag_{2.0}$@r–TiO_2/g–C_3N_4) in deionized H_2O

**Photocatalysts ($Ag_{2.0}$@r–TiO_2/g–C_3N_4)**	**Time (h)**						**H_2 generation**
	1	2	3	4	5	6	
1st run	14.812	19.007	23.831	29.319	35.179	**39.78**	mmol.g^{-1}
2nd run	12.117	16.998	20.716	24.336	29.163	33.726	mmol.g^{-1}
3rd run	10.031	13.119	16.415	20.194	24.409	28.234	mmol.g^{-1}
4th run	8.990	11.96	14.318	17.319	21.001	24.110	mmol.g^{-1}
5th run	6.192	9.027	12.032	15.423	19.181	22.333	mmol.g^{-1}

Table. (S2). Durability test for the high performing photocatalysts ($Ag_{2.0}$@r–TiO_2/g–C_3N_4) in Arabian seawater

**Photocatalysts ($Ag_{2.0}$@r–TiO_2/g–C_3N_4)**	**Time (h)**						**H_2 generation**
	1	2	3	4	5	6	
1st run	38.877	44.138	51.874	57.781	64.839	70.56	mmol.g^{-1}
2nd run	32.766	38.901	44.702	51.300	56.191	61.669	mmol.g^{-1}
3rd run	26.071	32.161	36.403	42.162	49.117	54.109	mmol.g^{-1}
4th run	23.140	29.006	33.121	38.307	43.139	47.110	mmol.g^{-1}
5th run	16.148	20.131	25.062	29.182	34.161	39.333	mmol.g^{-1}

Table. (S3). Experimental hydrogen generation activities by $Ag_{2.0}$@r–TiO_2/g–C_3N_4 (most active photocatalysts) at different concentration dose.

Sr. No.	$Ag_{2.0}$@r–TiO_2/g–C_3N_4 Vs. dose (mg)	H_2 evolution (mmol g^{-1} h^{-1})	
		**Deionized-H_2O**	**Seawater**
**1.**	1mg	1.921 ± 0.096	2.521 ± 0.126
**2.**	2mg	3.999 ± 0.1999	5.007 ± 0.2503
**3.**	3mg	4.314 ± 0.2157	8.163 ± 0.408
**4.**	4mg	6.63 ± 0.3315	11.76 ± 0.588
**5.**	5mg	5.203 ± 0.2601	9.125 ± 0.456
**6.**	6mg	4.871 ± 0.243	7.001 ± 0.350
**7.**	7mg	3.712 ± 0.185	5.909 ± 0.295
**8.**	8mg	2.61 ± 0.1305	3.991 ± 0.199

Optimized system includes; concentrated sunlight irradiations, pH = 8 and temperature = 35°C

Table. (S4). Experimental hydrogen generation activities by $Ag_{2.0}$@r–TiO_2/g–C_3N_4 (most active photocatalysts) at different pH.

Sr. No.	$Ag_{2.0}$@r–TiO_2/g–C_3N_4 vs. (pH)	H_2 evolution (mmol g^{-1} h^{-1})	
		**Deionized-H_2O**	**Seawater**
**1.**	1.0	1.847 ± 0.092	2.501 ± 0.125
**2.**	2.0	2.761 ± 0.138	3.234 ± 0.162
**3.**	3.0	3.623 ± 0.181	4.011 ± 0.200
**4.**	4.0	4.021 ± 0.201	5.509 ± 0.275
**5.**	5.0	5.021 ± 0.251	6.935 ± 0.346
**6.**	6.0	5.345 ± 0.422	8.743 ± 0.437
**7.**	7.0	6.212 ± 0.267	10.135 ± 0.506
**8.**	8.0	6.63 ± 0.33	11.760 ± 0.588
**9.**	9.0	6.011± 0.301	11.001 ± 0.550
**10.**	10.0	5.112 ± 0.255	9.213 ± 0.4606

Optimized system includes; concentrated sunlight irradiations, 4mg does of photocatalysts, pH = 8 and temperature = 35°C

Table. (S5). Experimental hydrogen generation activities by $Ag_{2.0}$@r–TiO_2/g–C_3N_4 (most active photocatalysts) at different intensities of sunlight.

Sr. No.	$Ag_{2.0}$@r–TiO_2/g–C_3N_4 vs. light intensity	H_2 evolution (mmol g^{-1} h^{-1})	
		Deionized-H_2O	Seawater
**1.**	10:00 AM-11.00 AM	3.201 ± 0.160	5.029 ± 0.251
**2.**	11:00 AM-12:00 PM	4.390 ± 0.219	7.452 ± 0.372
**3.**	12:00 AM-1:00 PM	5.441 ± 0.272	9.379 ± 0.468
**4.**	1:00 PM-2:00 PM	6.630 ± 0.331	11.76 ± 0.588
**5.**	2:00 AM-3:00 PM	6.011 ± 0.300	10.439 ± 0.521
**6.**	3:00 PM-4:00 PM	4.072 ± 0.203	8.3030 ± 0.415

Optimized system includes; 4mg does of photocatalysts; pH = 8 and temperature = 35°C

Table. (S6). Experimental hydrogen generation activities by $Ag_{2.0}$@r–TiO_2/g–C_3N_4 (most active photocatalysts) at different temperature.

Sr. No.	$Ag_{2.0}$@r–TiO_2/g–C_3N_4 vs. °C	H_2 evolution (mmol g^{-1} h^{-1})	
		Deionized-H_2O	Seawater
**1.**	25	2.503 ± 0.125	7.993 ± 0.399
**2.**	30	4.807 ± 0.240	9.103 ± 0.455
**3.**	35	6.63 ± 0.331	11.76 ± 0.588
**4.**	40	5.417 ± 0.270	10.04 ± 0.502
**5.**	45	4.437 ± 0.221	9.031 ± 0.452
**6.**	50	3.002 ± 0.150	7.20 ± 0.360

Optimized system includes; concentrated sunlight irradiations, 4mg does of photocatalysts; pH = 8

Section#3

Table. (S7). Comparison of rate of H_2 evolution ($mmol.g^{-1}h^{-1}$) of current studies with g-C_3N_4 and TiO_2 based photocatalysts with reported studies.

Photocatalyst	Technique	h^+ scavenger	H_2O	H_P ($mmol.g^{-1}h^{-1}$)	E.F*	Ref.
Meso-B/A	Hydrothermal	Absent	S.W	6.59	2.5 > B/R TiO_2	[1]
HCCN/CAN	Solvothermal	TEOA	S.W	3.147	6.8 > ACN	[2]
Hollow tubular g-C_3N_4	Hydrothermal	TEOA	DI-W	8.683	19.3 > BCN	[3]
$Mn_{0.8}Cd_{0.2}S$/g-C_3N_4	Hydrothermal	S_2^-, SO_3^{2-}	DI-W	4.0	3.4> $Mn_{0.8}Cd_{0.2}S$	[4]
CuO/pCN	Thermal polymerization	Methanol	DI-W	0.030	2.78 > pure CN	[5]
α-Fe_2O_3/g-C_3N_4	Thermal etching	TEOA	DI-W	5.0	1.67 > BCN	[6]
Ru/TP-Ru/EC_3N_4	Exfoliation	Methanol	DI-W	2.562	48.81 > g-C_3N_4	[7]
M-CA- g-C_3N_4	Ball milling	Ethanol	DI-W	0.74	5 > g-C_3N_4	[8]
$Ag_{2.0}$@r–TiO_2/g–C_3N_4	Hydrothermal	Lactic acid	DI-W	6.63	9.56 and 6.21> g-C_3N_4 and r–TiO_2	**This work**
$Ag_{2.0}$@r–TiO_2/g–C_3N_4	Hydrothermal	Lactic acid	S.W	11.76	37.94 and 14.16> g-C_3N_4 and r–TiO_2	**This work**

E.F*= enhancement factor; H_P = hydrogen production; DI-W = deionized water; S.W = seawater

References

1. Z. Cheng, X. Zhang, C. Bo, Y. Sun, C. Li and L. Piao, *International Journal of Hydrogen Energy*, 2024, **55**, 542-549.
2. H. Sun, Y. Shi, W. Shi and F. Guo, *Applied Surface Science*, 2022, **593**, 153281.
3. F. Guo, Z. Chen, Y. Shi, L. Cao, X. Cheng, W. Shi, L. Chen and X. Lin, *Renewable Energy*, 2022, **188**, 1-10.
4. H. Liu, Z. Xu, Z. Zhang and D. Ao, *Applied Catalysis A: General*, 2016, **518**, 150-157.
5. A. Raza, A. A. Haidry, T. Amin, A. A. Hussain, S. A. M. H. Shah and M. Ahsan, *Diamond and Related Materials*, 2024, **141**, 110703.
6. Y. Li, S. Zhu, Y. Liang, Z. Li, S. Wu, C. Chang, S. Luo and Z. Cui, *Materials & Design*, 2020, **196**, 109191.
7. M. Tahir, *Energy & Fuels*, 2024, **38**, 14588-14603.
8. B. Arkhurst, R. Guo, D. Gunawan, L. Oppong-Antwi, A. N. Ashong, X. Fan, G. B. Rokh and S. L. I. Chan, *International Journal of Hydrogen Energy*, 2024, **87**, 321-331.
